# Dietary and lifestyle oxidative balance scores and their impact on cardiovascular diseases: exploring the mediating influence of hepatic function and blood lipid levels

**DOI:** 10.3389/fcvm.2025.1493271

**Published:** 2025-02-19

**Authors:** Yi He, Ying Lan

**Affiliations:** ^1^ Third Military Medical University (Army Medical University), Chongqing, China; ^2^ Department of Critical Care Medicine, Affiliated Hospital of Chengdu University, Chengdu, Sichuan, China

**Keywords:** cardiovascular diseases, heart failure, hepatic function, lipid level, oxidative balance score

## Abstract

**Background:**

Limited evidence exists regarding the association between oxidative stress induced by dietary and lifestyle factors and cardiovascular diseases (CVDs).

**Methods:**

We conducted a weighted analysis using data from 13,530 adults in the National Health and Nutrition Examination Survey (NHANES), covering the period from 2003 to 2018. The total oxidative balance score (OBS) was derived from 20 oxidative stress-related exposures, including dietary and lifestyle factors. Survey-weighted multivariable logistic regression and stratified analyses were performed to examine the association between OBS and CVDs. To further investigate the nonlinear relationship, we employed restricted cubic spline analysis and threshold effect analysis. Additionally, we assessed whether hepatic function and blood lipid levels mediate the OBS-CVDs relationship.

**Results:**

This study included 13,530 participants, representing a weighted population of 81,006,649.2 individuals. After adjusting for potential confounders, a linear relationship was observed between total OBS, dietary OBS, and CVDs, while lifestyle OBS exhibited a nonlinear association, with a significant threshold effect at a score of 4. When the lifestyle OBS exceeded this threshold, a marked negative correlation with CVDs was observed. Furthermore, albumin (Alb) mediated 23.59% of the relationship between the total OBS and CVDs.

**Conclusions:**

Lower levels of total OBS were inversely associated with CVDs. This association was partially mediated by blood lipid levels and hepatic function. Interventions focusing on antioxidant-rich dietary and lifestyle modifications may play a pivotal role in reducing the possibility of CVDs.

## Introduction

Oxidative stress results from the accumulation of reactive oxygen species (ROS), which can damage cellular structures and disrupt their functions. When the body's antioxidant defenses are overwhelmed by an excess of free radicals, oxidative stress develops, potentially leading to damage to proteins, lipids, and DNA, and contributing to the pathogenesis of various diseases (1). Prolonged exposure to ultraviolet radiation, chronic stress, intense physical activity, unhealthy dietary habits, and exposure to irritants can lead to excessive production of ROS (2). Unhealthy lifestyle behaviors are significant contributors to the global prevalence of cardiovascular diseases (CVDs). Smoking, alcohol consumption, excess body weight, high fat intake, and iron are potential pro-oxidants, while dietary antioxidants such as fiber, carotene, riboflavin, certain vitamins, and physical exercise, serve as common antioxidant factors. The Oxidative Balance Score (OBS) offers a new approach by integrating multiple dietary and lifestyle exposures, assessing an individual's overall antioxidant status by considering both antioxidant and pro-oxidant factors. A higher OBS reflects a greater exposure to antioxidants.

Previous studies have shown that a higher OBS is associated with reduced all-cause and cardiovascular mortality in individuals with diabetes and prediabetes (3). The mechanisms through which the OBS influences CVDs require further investigation. Evidence suggests that oxidative stress is closely associated with hepatotoxicity, disrupting the intracellular oxidative balance in liver cells and impairing both their structure and function (4). This disruption often leads to liver dysfunction, which is a well-established precursor to dyslipidemia (5, 6). Building on this understanding, we hypothesize that low OBS levels may exacerbate oxidative stress, potentially affecting serum markers such as albumin (Alb), total cholesterol (TC), and alkaline phosphatase (ALP), thereby increasing the possibility of developing CVDs.

To address these concerns, we analyzed cross-sectional data from eight cycles of the National Health and Nutrition Examination Survey (NHANES), including 13,530 participants, to examine the relationship between OBS and both general and specific CVDs. Additionally, this study aims to conduct a mediation analysis to explore whether the effects of OBS on CVDs are mediated through hepatic function and lipid levels, with the ultimate goal of providing novel intervention strategies for clinical application.

## Methods

### Study population

The NHANES is an ongoing cross-sectional survey designed to assess the health and nutritional status of non-institutionalized civilians across the United States. Employing a complex multistage probability sampling design, NHANES ensures national representativeness. Participants undergo household interviews, physical examinations, and laboratory tests every two years. The survey is administered by the National Center for Health Statistics (NCHS), which operates under the Centers for Disease Control and Prevention (CDC). All participants provide written informed consent prior to participation.

**Figure 1:**
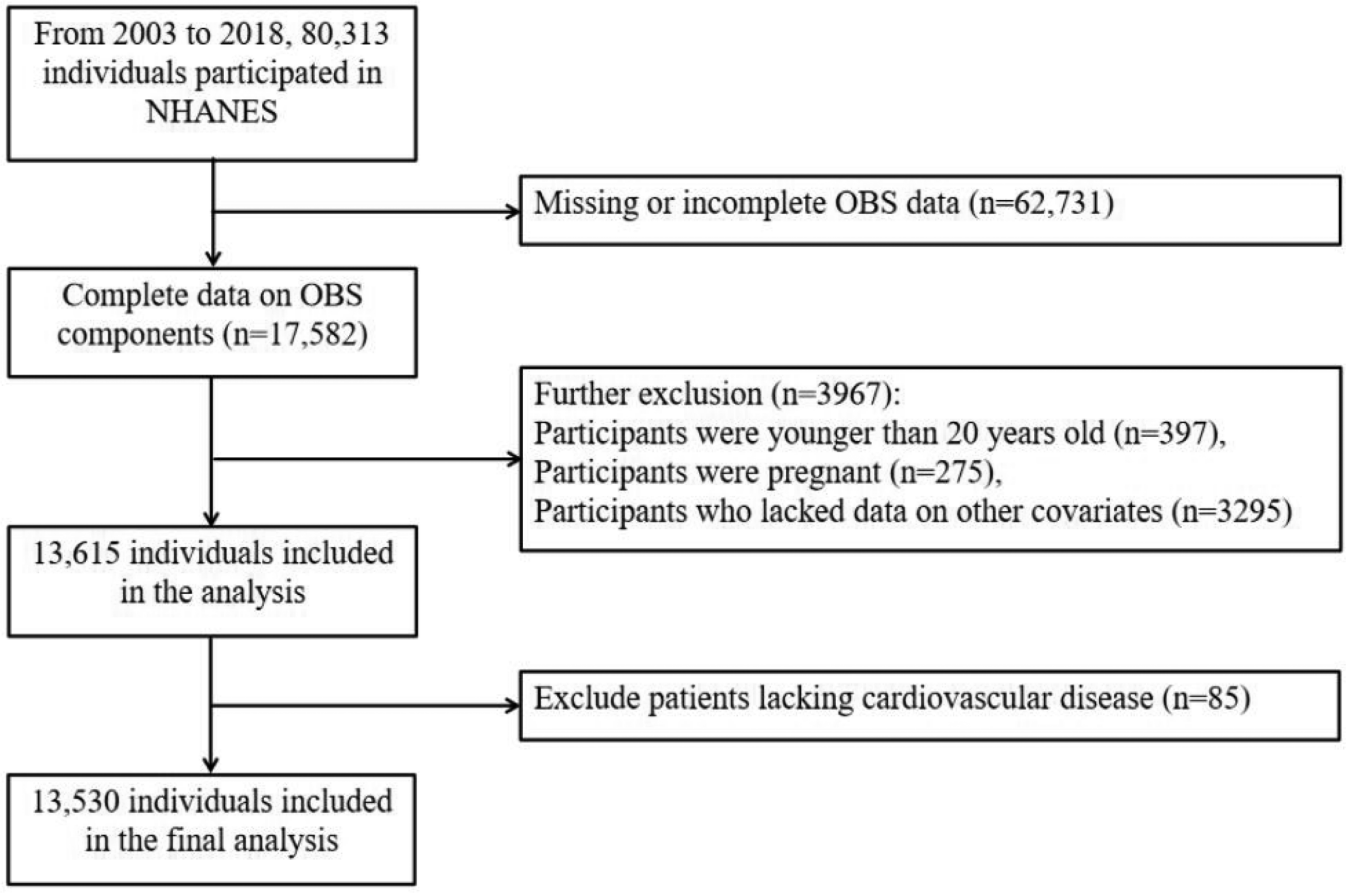
Flowchart of the study population selection for final analysis.

This study utilized publicly available NHANES data from 2003 to 2018 and initially included 80,313 adults. A total of 67,708 individuals were excluded from the analysis for various reasons: 62,731 had incomplete OBS data, 397 were under the age of 20, 275 were pregnant women, 3,295 had missing covariate data, and another group had unreliable energy intake values (less than 800 kcal/day or more than 4,200 kcal/day for males, and less than 500 kcal/day or more than 3,500 kcal/day for females). After excluding 85 individuals with missing data on CVDs, the final analysis included 13,530 participants with complete interview and examination data (Figure 1). Sociodemographic information was collected, including age, gender, race, education levels, poverty-income ratio (PIR), and body mass index (BMI). Additionally, covariates such as total energy intake, blood lipid levels, liver and kidney function indicators, and self-reported chronic conditions, including diabetes and hypertension, were gathered (3).

### Oxidative balance score

The OBS is a comprehensive metric that represents the overall oxidative balance, derived from 16 dietary nutrients and 4 lifestyle components, encompassing both pro-oxidant and antioxidant factors. Pro-oxidant factors include total fat and iron intake, smoking and alcohol habits, and BMI, while antioxidant factors include dietary fiber, calcium, zinc, copper, selenium, magnesium, vitamins C, E, B12, B6, carotenoids, riboflavin, niacin, total folate intake, and physical activity. Each component was categorized into three groups based on gender-specific tertiles. Antioxidant components in Groups 1 to 3 were assigned scores from 0 to 2, with higher scores indicating greater antioxidant levels. Conversely, pro-oxidant components were scored inversely, with lower scores indicating higher pro-oxidant levels. Specific scoring for each component is as follows:

- Physical activity levels were categorized based on the 2018 Physical Activity Guidelines for Americans (HHS, 2018): low activity (<150 min per week) was assigned a score of 0, moderate activity (150–300 min per week) was assigned a score of 1, and high activity (>300 min per week) was assigned a score of 2.
- Alcohol consumption was classified as follows: non-drinkers were assigned a score of 2, moderate drinkers (0–15 g per day for females or 0–30 g per day for males) were assigned a score of 1, and heavy drinkers (≥15 g per day for females or ≥30 g per day for males) were assigned a score of 0.
- Smoking status was assessed based on lifetime cigarette consumption: individuals who smoked fewer than 100 cigarettes scored 2, those with at least 100 cigarettes but who were no longer smoking scored 1, and current smokers scored 0.
- BMI was calculated as weight (kg) divided by height squared (m^2^): individuals with normal weight (<25 kg/m^2^) received a score of 2, those who were overweight (25–29.9 kg/m^2^) received a score of 1, and individuals with obesity (≥30 kg/m^2^) received a score of 0.

The dietary OBS was obtained by totaling the scores of the 16 dietary components, while the lifestyle OBS was determined by summing the scores of the 4 lifestyle components. The total OBS was obtained by adding together the scores of each individual component. In our investigation, total OBS ranged from 5 to 38, with higher scores denoting greater levels of antioxidant exposure. Participants were stratified into quartiles according to their total OBS, with Quartiles 1 and 2 categorized as pro-oxidant groups, and Quartiles 3 and 4 classified as antioxidant groups.

### Outcome definitions

The diagnosis of CVDs was established through a comprehensive medical history questionnaire conducted via individual interviews. This questionnaire comprised five distinct inquiries, such as: “Has a doctor or other healthcare professional ever informed you that you have congestive heart failure (HF)/coronary heart disease (CHD)/angina/heart attack/stroke?” An affirmative response to any of these queries indicated a positive CVDs status for the individual. Participants who provided affirmative answers to specific CVD-related questions were categorized accordingly.

### Statistical analysis

To assess the relationship between OBS and CVDs, survey-weighted multivariable logistic regression analysis was performed. Given the complex sampling design of NHANES, sample data were weighted using the “survey” package in R. To account for the eight cycles of NHANES, the original two-year sample weights were divided by eight and assigned to each participant. Subsequent regression analyses utilized these weighted models. Three covariate-adjusted models were employed to assess potential confounding effects. Stratified analyses were performed to evaluate the robustness of the relationship between OBS and CVDs. Dose-response associations were explored using restricted cubic splines with a smoothing function. Additionally, mediation analyses were conducted using the “mediation” package in R to estimate the potential mediating effects of TC, Alb, ALP on the OBS-CVDs association. Statistical analyses were performed using R software (version 4.3.2), with statistical significance set at *P* < 0.05.

## Results

### Population characteristics

This study involved 13,530 participants, weighted to represent a population of 81,006,649.2 individuals. The mean age was 45.77 ± 16.19 years, comprising 41,673,433.2 males (51.4%) and 39,333,216.0 females (48.6%). Among the participants, 5,031,828.7 individuals (6.2%) were diagnosed with CVDs (Table 1). Individuals with CVDs tended to be male, older, non-Hispanic white, and exhibited higher BMI, hypertension, lower levels of TC, HDL, Alb, and calorie intake, while showing elevated levels of ALP and creatinine. Notably, CVDs patients had lower total, lifestyle, and dietary OBS.

**Table 1:** Survey-weighted baseline characteristics of the U.S. Adult Population, NHANES 2003–2018 Cohort.

	Levels	Overall	Non-CVDs	CVDs	*P*
N		81006649.2	75974820.5	5031828.7	
Gender (%)	Male	41673433.2 (51.4)	38643989.9 (50.9)	3029443.4 (60.2)	<0.001
	Female	39333216.0 (48.6)	37330830.7 (49.1)	2002385.3 (39.8)	
Age [mean (SD)]		45.77 (16.19)	44.59 (15.67)	63.60 (13.19)	<0.001
Race (%)	Mexican American	5703257.4 (7.0)	5520859.6 (7.3)	182397.9 (3.6)	<0.001
	Other Hispanic	3937959.1 (4.9)	3790056.0 (5.0)	147903.1 (2.9)	
	Non-Hispanic White	58386889.0 (72.1)	54470969.1 (71.7)	3915919.9 (77.8)	
	Non-Hispanic Black	7234802.3 (8.9)	6790057.4 (8.9)	444744.9 (8.8)	
	Other Race	5743741.4 (7.1)	5402878.5 (7.1)	340862.9 (6.8)	
Education (%)	Less than 9th Grade	2464750.5 (3.0)	2224464.9 (2.9)	240285.6 (4.8)	<0.001
	9–11th Grade	5963156.3 (7.4)	5488192.0 (7.2)	474964.3 (9.4)	
	High school Grad/GED	17358471.2 (21.4)	16059375.9 (21.1)	1299095.4 (25.8)	
	Some college or AA degree	25732196.0 (31.8)	24136857.9 (31.8)	1595338.0 (31.7)	
	College Graduate or above	29488075.2 (36.4)	28065929.8 (36.9)	1422145.4 (28.3)	
PIR [mean (SD)]		3.24 (1.62)	3.24 (1.62)	3.12 (1.62)	0.111
HF (%)	No	79910832.8 (98.6)	75974820.5 (100.0)	3936012.3 (78.2)	<0.001
	Yes	1095816.4 (1.4)	0.0 (0.0)	1095816.4 (21.8)	
CHD (%)	No	78734833.7 (97.2)	75974820.5 (100.0)	2760013.2 (54.9)	<0.001
	Yes	2271815.5 (2.8)	0.0 (0.0)	2271815.5 (45.1)	
Angina (%)	No	79672305.4 (98.4)	75974820.5 (100.0)	3697484.9 (73.5)	<0.001
	Yes	1334343.8 (1.6)	0.0 (0.0)	1334343.8 (26.5)	
Heart attack (%)	No	79009979.0 (97.5)	75974820.5 (100.0)	3035158.5 (60.3)	<0.001
	Yes	1996670.2 (2.5)	0.0 (0.0)	1996670.2 (39.7)	
Stroke (%)	No	79578794.5 (98.2)	75974820.5 (100.0)	3603974.0 (71.6)	<0.001
	Yes	1427854.7 (1.8)	0.0 (0.0)	1427854.7 (28.4)	
Hypertension (%)	No	58752938.1 (72.5)	57016904.7 (75.0)	1736033.4 (34.5)	<0.001
	Yes	22253711.1 (27.5)	18957915.8 (25.0)	3295795.3 (65.5)	
Diabetes (%)	No	75074338.0 (92.7)	71226079.4 (93.7)	3848258.6 (76.5)	<0.001
	Yes	5932311.2 (7.3)	4748741.1 (6.3)	1183570.1 (23.5)	
BMI [mean (SD)]		28.41 (6.40)	28.31 (6.36)	29.98 (6.80)	<0.001
TC [mean (SD)]		5.03 (1.07)	5.06 (1.05)	4.64 (1.15)	<0.001
HDL [mean (SD)]		1.41 (0.43)	1.42 (0.43)	1.34 (0.44)	<0.001
Alb [mean (SD)]		43.06 (3.24)	43.14 (3.23)	41.78 (3.13)	<0.001
ALT [mean (SD)]		25.39 (18.21)	25.39 (18.15)	25.40 (19.14)	0.996
AST [mean (SD)]		25.30 (16.07)	25.26 (16.20)	25.83 (13.95)	0.259
ALP [mean (SD)]		65.95 (21.73)	65.52 (21.42)	72.46 (25.13)	<0.001
Creatinine [mean (SD)]		78.05 (24.45)	77.24 (22.45)	90.20 (43.07)	<0.001
Calorie [mean (SD)]		2133.52 (774.95)	2145.79 (776.85)	1948.31 (721.16)	<0.001
Lifestyle OBS [mean (SD)]		5.29 (1.53)	5.31 (1.54)	4.99 (1.43)	<0.001
Dietary OBS [mean (SD)]		16.59 (6.56)	16.66 (6.56)	15.57 (6.48)	<0.001
Total OBS [mean (SD)]		21.88 (6.84)	21.96 (6.84)	20.55 (6.75)	<0.001
Total OBS category (%)	Q1	11194831.0 (13.8)	10276489.2 (13.5)	918341.8 (18.3)	<0.001
	Q2	26412731.9 (32.6)	24582595.1 (32.4)	1830136.8 (36.4)	
	Q3	31364091.7 (38.7)	29607335.4 (39.0)	1756756.3 (34.9)	
	Q4	12034994.6 (14.9)	11508400.8 (15.1)	526593.8 (10.5)	

PIR, poverty-income ratio; HF, heart failure; CHD, coronary heart disease; CVDs, cardiovascular diseases; BMI, body mass index; TC, total cholesterol; HDL, high density lipoprotein; Alb, albumin; ALT, alanine aminotransferase; AST, aspartate aminotransferase; ALP, alkaline phosphatase; OBS, oxidative balance score.

### Associations between OBS and CVDs

**Figure 2:**
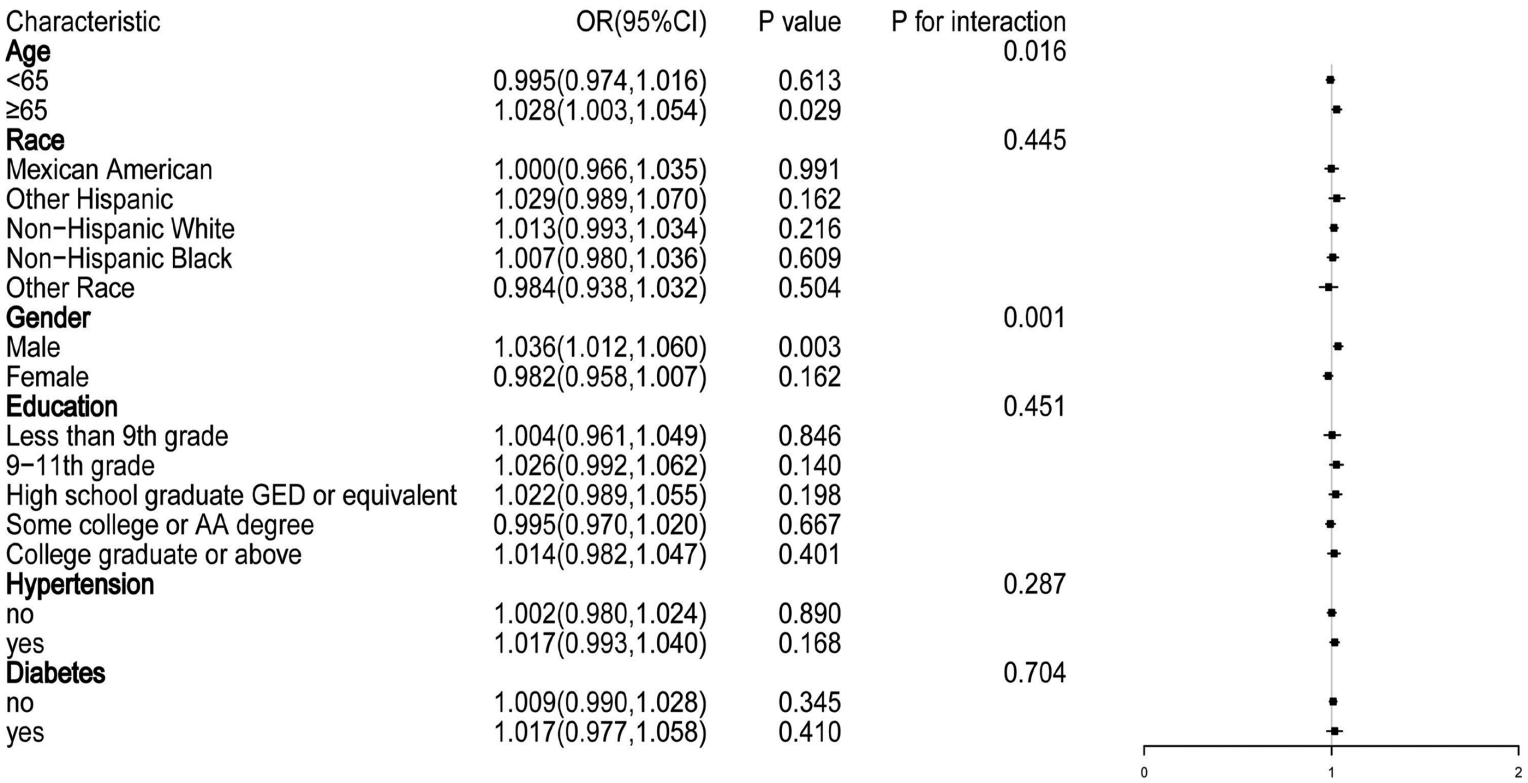
Subgroup analysis of the relationship between total OBS and CVDs. Adjusted for age, gender, education levels, race, hypertension, diabetes, TC, Alb, ALP, creatinine, and caloric intake.

Table 2 presents the association between total OBS and CVDs as determined by logistic regression analysis. After adjusting for potential confounders, a significant association was observed. In Model 3, participants in the highest total OBS Quartile had a reduced likelihood of CVDs occurrence (Q4 vs. Q1: OR=−0.51; 95%CI = −0.89 to −0.12, *P* < 0.01). Similarly, individuals in the third total OBS Quartile also showed a lower likelihood of developing CVDs, with an OR of −0.34 (95%CI = −0.60 to −0.09; *P* < 0.01). Further investigation revealed a significant negative correlation between total OBS subgroups and HF, whereas no statistically significant associations were detected with CHD, angina pectoris, heart attack, or stroke. Subgroup analysis (Figure 2) comparing individuals with and without CVDs indicated that age and gender modulated the OBS-CVDs relationship. That is to say, this relationship was more prominent in elderly and male patient groups (*P* < 0.05).

**Table 2:** Weighted multivariate logistic regression analysis of the association between OBS and overall CVDs and specific CVD subtypes.

		Model 1	Model 2	Model 3
		OR (95%CI), *P*	OR (95%CI), *P*	OR (95%CI), *P*
CVDs	Q1	Ref	Ref	Ref
	Q2	−0.18(−0.40, 0.04), *P* = 0.1	−0.21(−0.45, 0.02), *P* = 0.07	−0.18(−0.42, 0.07), *P* = 0.15
	Q3	−0.41(−0.63, −0.19), *P* < 0.01	−0.43(−0.66, −0.19), *P* < 0.01	−0.34(−0.60, −0.09), *P* < 0.01
	Q4	−0.67(−1.00, −0.34), *P* < 0.01	−0.63(−0.99, −0.26), *P* < 0.01	−0.51 (−0.89, −0.12), *P* < 0.01
HF	Q1	Ref	Ref	Ref
	Q2	−0.59(−1.01, −0.18), *P* < 0.01	−0.56(−1.01, −0.11), *P* = 0.02	−0.50(−0.95, −0.05), *P* = 0.03
	Q3	−0.97(−1.42, −0.53), *P* < 0.01	−0.86(−1.32, −0.41), *P* < 0.01	−0.71(−1.19, −0.23), *P* < 0.01
	Q4	−1.41(−2.17, −0.65), *P* < 0.01	−1.21(−1.97, −0.45), *P* < 0.01	−0.95(−1.72, −0.18), *P* = 0.02
Angina	Q1	Ref	Ref	Ref
	Q2	−0.06(−0.56, 0.44), *P* = 0.81	−0.06(−0.58, 0.46), *P* = 0.81	−0.02(−0.54, 0.51), *P* = 0.95
	Q3	−0.38(−0.84, 0.07), *P* = 0.10	−0.36(−0.85, 0.12), *P* = 0.14	−0.26(−0.77, 0.25), *P* = 0.32
	Q4	−0.54(−1.27, 0.19), *P* = 0.15	−0.45(−1.20, 0.31), *P* = 0.25	−0.29(−1.06, 0.48), *P* = 0.46
Heart attack	Q1	Ref	Ref	Ref
	Q2	−0.17(−0.54, 0.19), *P* = 0.35	−0.20(−0.62, 0.22), *P* = 0.35	−0.14 (0.58, 0.30), *P* = 0.53
	Q3	−0.33(−0.69, 0.04), *P* = 0.08	−0.33(−0.74, 0.09), *P* = 0.12	−0.21(−0.64, 0.22), *P* = 0.33
	Q4	−0.85(−1.45, −0.25), *P* < 0.01	−0.77(−1.42, −0.12), *P* < 0.05	−0.61(−1.28, 0.05), *P* = 0.07
Stroke	Q1	Ref	Ref	Ref
	Q2	0.10(−0.32, 0.52), *P* = 0.62	0.19(−0.23, 0.61), *P* = 0.37	0.20(−0.23, 0.63), *P* = 0.37
	Q3	−0.36(−0.74, 0.02), *P* = 0.07	−0.21(−0.62, 0.20), *P* = 0.31	−0.16(−0.59, 0.27), *P* = 0.46
	Q4	−0.53(−1.12, 0.07), *P* = 0.08	−0.31(−0.93, 0.31), *P* = 0.32	−0.26(−0.91, 0.40), *P* = 0.44
CHD	Q1	Ref	Ref	Ref
	Q2	−0.26(−0.62, 0.10), *P* = 0.15	−0.40(−0.81, 0.01), *P* = 0.05	−0.35(−0.78, 0.07), *P* = 0.10
	Q3	−0.25(−0.61, 0.10), *P* = 0.16	−0.40(−0.80, 0.01), *P* = 0.05	−0.30(−0.74, 0.14), *P* = 0.18
	Q4	−0.55(−1.04, −0.06), *P* < 0.05	−0.63(−1.19, −0.07), *P* = 0.03	−0.53(−1.11, 0.05), *P* = 0.07

CI, confidence interval; CVDs, cardiovascular diseases; OBS, oxidative balance score; OR, odds ratio; Ref, reference; Q – quartile.

Model 1: Crude.

Model 2: Adjusted for age, gender, race, PIR.

Model 3: Adjusted for age, gender, race, PIR, hypertension, diabetes, BMI, TC, Alb, ALP, creatinine.

**Figure 3:**
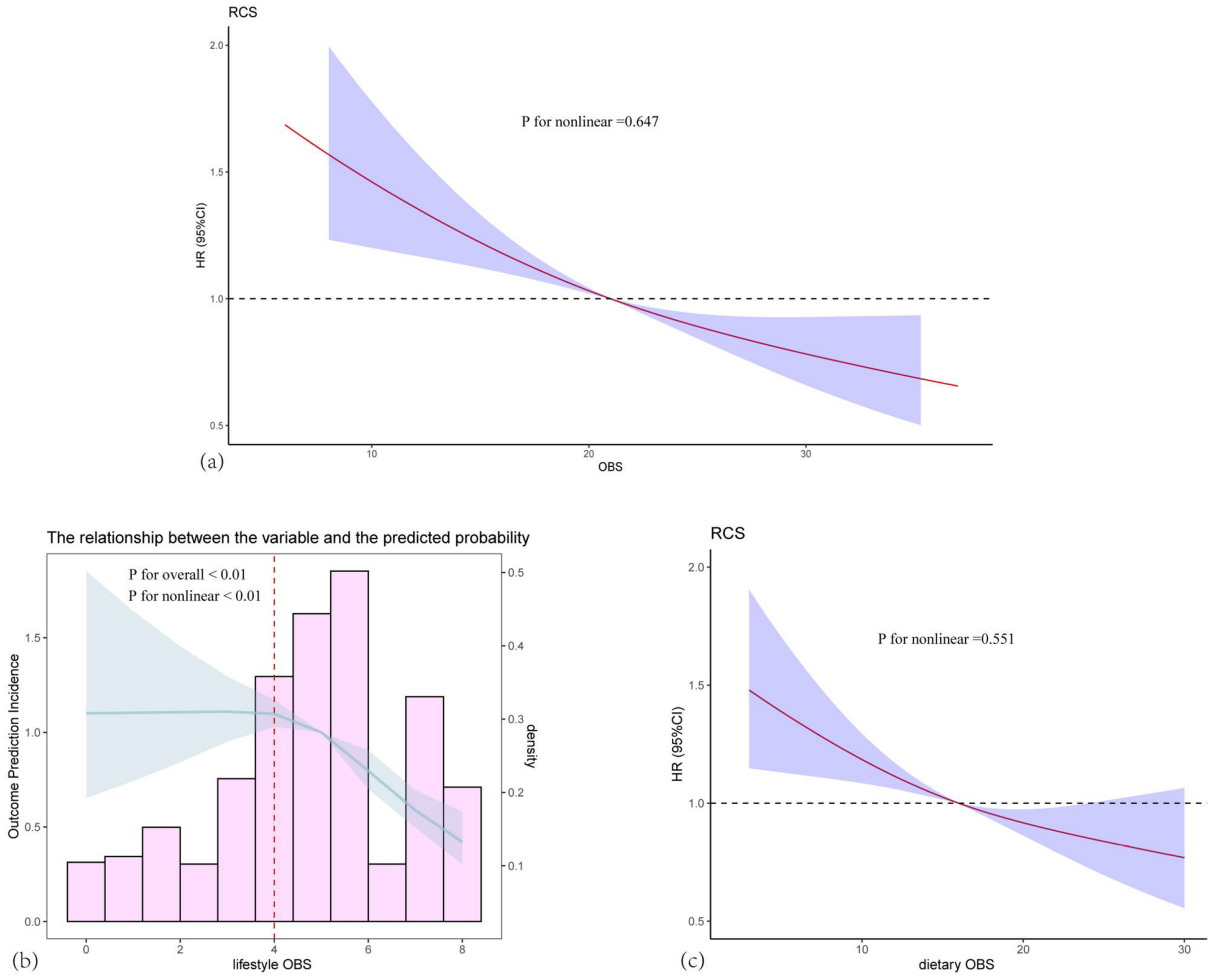
Dose-Response associations between total OBS, dietary OBS, and lifestyle OBS and CVDs. **(a)** Total OBS; **(b)** Lifestyle OBS; **(c)** Dietary OBS. The Solid lines and shaded areas represent the central risk estimates and 95% CIs. Logistic regression based on restricted cubic splines shows a linear association between dietary OBS and CVDs (*P* for nonlinearity = 0.647), and a nonlinear association between lifestyle OBS and CVDs (*P* for nonlinearity < 0.01). Models were adjusted for age, gender, race, and education levels.

The restricted cubic spline curves revealed a linear trend in the relationship between total OBS (*P* for non-linearity = 0.647) and dietary OBS (*P* for non-linearity = 0.551) with CVDs, as illustrated in Figure 3. Specifically, the association between lifestyle OBS and CVDs exhibited a non-linear trend (*P* for non-linearity < 0.01; Table 3). Threshold effect analysis indicated that when lifestyle OBS was below 4, no statistically significant association was observed. However, when lifestyle OBS exceeded 4, a significant negative correlation emerged, with an OR of 0.813 (*P* < 0.01). A likelihood ratio test comparing segmented models with linear models yielded a *P* = 0.014, suggesting that a segmented model with a significant turning point at lifestyle OBS = 4 was more appropriate.

**Table 3:** Threshold effect of lifestyle OBS on CVDs analyzed using a Two-stage logistic regression model.

Lifestyle OBS	Adjusted OR(95%), *P*
Model 1 Fitting model by standard linear regression	0.86 (0.82, 0.90), *P* < 0.01
Model 2 Fitting model by two-piecewise linear regression	
Inflection point	4
<4	1.00 (0.88, 1.15), *P* = 0.988
>4	0.81 (0.76, 0.87), *P* < 0.01
*P* for likelihood ratio test	*P* = 0.014

### Mediation analyses with separate mediators between OBS and CVDs

We further examined whether liver function-related markers mediate the negative correlation between OBS and CVDs. Based on the significant results from multivariable logistic regression analysis, we selected Alb, TC, and ALP as the variables for mediation analysis. As depicted in Table 4, Alb mediated 23.59% of the association between OBS and CVDs, TC mediated −8.29%, and ALP mediated 7.68% (all *P* < 0.05).

**Table 4:** Estimated proportion of the association between tital OBS and CVDs mediated by Serum parameters.

	Total Effect（95%CI)	*P*	Direct effect（95%CI)	*P*	Indirect effect（95%CI)	*P*	Proportion of mediation
TC	−1.02E-03(−2.07E-03, 0.00)	<0.01	−1.10E-03 (−2.17e-03, 0.00)	<0.01	7.90E-05 (3.00E-05, 0.00)	0.02	−8.29%
Alb	−1.537E-03(−2.732E-03, 0.00)	<0.01	−1.10E-03 (−2.328E-03, 0.00)	0.02	−3.51E-04(−4.73E-04, 0.00)	<0.01	23.59%
ALP	−1.15E-03(−2.21E-03, 0.00)	<0.01	−1.06E-03 (−2.13E-03, 0.00)	<0.01	−9.04E-05(−1.56E-04, 0.00)	<0.01	7.68%

All analyses were adjusted for age, gender, education, race, hypertension, diabetes, Alb, ALP, creatinine, calorie intake.

## Discussions

In this nationwide prospective cohort study utilizing NHANES data spanning up to 16 years, we observed a negative correlation between total OBS and CVDs. Participants with a pro-oxidative OBS exhibited a significantly higher possibility of overall CVDs, with a particularly pronounced association observed in HF. Subgroup analysis revealed a more pronounced correlation in elderly and male patients (*P* < 0.05). A linear relationship was identified between total and dietary OBS with CVDs, while lifestyle OBS exhibited a non-linear relationship. Notably, Alb played a significant mediating role in this association.

While the precise mechanism by which OBS affects CVDs remains unclear, oxidative stress appears to play a central role in this association. ROS are oxygen-containing molecules characterized by their chemical reactivity, functioning both as signaling molecules and as harmful agents when present in excess. Excessive accumulation of ROS within cells can trigger cellular stress responses, including the activation of antioxidant defense mechanisms or programmed cell death pathways (7). OBS may exacerbate CVDs progression by promoting ROS production. Atherosclerosis, with its multifactorial etiology, highlights the central role of inflammation (8). From endothelial dysfunction to clinical manifestations, both systemic and local inflammation play pivotal roles in the onset and progression of CVDs. Monocytes recruited to sites of inflammation differentiate into macrophages, which exhibit either pro-inflammatory or anti-inflammatory phenotypes based on microenvironment cues (9).

The linear relationship between total and dietary OBS with CVDs suggests that dietary antioxidant status may have a predictable impact on cardiovascular health. This finding could inform dietary guidelines or interventions aimed at reducing CVDs possibility by emphasizing antioxidant-rich diets. In contrast, the non-linear relationship observed with lifestyle OBS indicates that lifestyle factors may influence CVDs in a more complex manner, suggesting the need for personalized lifestyle modifications rather than a one-size-fits-all approach. When lifestyle OBS is below the threshold of 4, changes in lifestyle OBS may not significantly impact CVDs. However, once lifestyle OBS exceeds this threshold, a notable reduction in CVDs possibility is observed, suggesting that specific lifestyle factors may have a more pronounced effect on CVDs prevention once they reach a certain level. Clinicians should consider assessing patients' total, dietary, and lifestyle OBS and tailor their recommendations accordingly.

Our study further explored the pathways linking OBS to CVDs through mediation analysis, identifying Alb, TC, and ALP as partial mediators. Alb alone accounted for 23.59% of the observed association, suggesting that reduced OBS may worsen CVDs symptoms by promoting oxidative stress and affecting lipid levels and liver function. A study by Andreas Ronit et al. also found a strong, independent link between low Alb and an increased risk of CVDs, with hazard ratios ranging from 1.17 to 1.46 across different CVDs subtypes. Meta-analysis revealed a risk ratio of 1.85 for each 10 g/L decrease in Alb, partly due to Alb's role as a negative acute-phase reactant (10). The Mediterranean diet, rich in antioxidants and anti-inflammatory compounds, helps alleviate intracellular oxidative stress and consistently benefits overall health, well-being, and mental state. It is believed to modulate genes related to oxidative stress, contributing to its health advantages (11). Panagiotakos et al. reported a significant negative correlation between adherence to the Mediterranean diet and blood lipid levels (12). Additionally, oxidative stress is associated with hepatotoxicity. Diets high in methionine can induce hyperhomocysteinemia, leading to multi-organ damage and inflammation. Research by Derouiche F found that methionine supplementation correlates with oxidative stress and disrupts proteasome function, resulting in hepatotoxicity and inflammation, as evidenced by elevated liver enzymes, bilirubin, and decreased Alb levels (4).

Notably, total OBS demonstrates a strong negative correlation with HF, but not with other subtypes like CHD, angina, heart attack, or stroke. This disparity likely reflects the distinct pathophysiological mechanisms underlying each CVD subtype, suggesting that the effects of antioxidants are not uniform across diseases. Additionally, antioxidants, particularly at high concentrations or when interacting with other substances, may act as pro-oxidants (13). Several factors may contribute to this variation, including differing baseline oxidative stress levels in each disease, the context-dependent effects of antioxidants, and the potential disruption of oxidative balance when antioxidant levels become excessive (14). In the case of HF, the pathogenesis involves complex processes such as myocardial remodeling, impaired contractility, neurohormonal activation, inflammation, and oxidative stress, all regulated by various genes (15).

This study has several limitations. Firstly, patients' self-reported CVDs history is prone to recall and information biases, as well as misclassification, which could compromise the reliability of the results. Secondly, residual and unmeasured confounding factors may introduce bias into the analysis. Thirdly, the observational design of our study limits our ability to establish a causal relationship between OBS and CVDs. Fourthly, despite our large sample size, the participants are limited to voluntary U.S. residents, which raises concerns about the generalizability of our findings to other countries. Given these limitations, the results should be interpreted with caution, and further research is needed to confirm our findings.

## Conclusions

Total OBS is negatively associated with both overall CVDs and HF, with total and dietary OBS demonstrating a linear negative correlation with CVDs, while lifestyle OBS exhibits a nonlinear relationship. Interventions promoting antioxidant-rich diets and healthy lifestyles could significantly reduce the likelihood of CVDs. Furthermore, our findings suggest that blood lipids and liver function may mediate the association between OBS and CVDs. Prospective and experimental studies are needed to confirm these associations and elucidate the underlying mechanisms. We recommend that clinical practice and public health initiatives focus on promoting antioxidant-rich diets, such as the Mediterranean diet, to mitigate oxidative stress and enhance cardiovascular health. Regular monitoring of liver function and lipid profiles should be incorporated into routine check-ups, especially for high-risk individuals.

## Data Availability

The original contributions presented in the study are included in the article/Supplementary Material, further inquiries can be directed to the corresponding author.
